# Effects of lumbosacral and lumbopelvic fixation and fusion on hip joint motor function: a single-center prospective cohort study using gait analysis

**DOI:** 10.3389/fsurg.2025.1692024

**Published:** 2026-01-09

**Authors:** Fansheng Zhang, Shuyuan Bian, Shijia Gaxi, Xiaobin Tian, Bo Li, Tao Guo

**Affiliations:** 1 Medical School of Guizhou University, Guiyang, China; 2 Department of Orthopaedics, Quzhou City Traditional Chinese Medicine Hospital, Quzhou, China; 3 Guizhou University of Traditional Chinese Medicine, Guiyang, China; 4 Guizhou Medical University, Guiyang, China; 5 Department of Orthopaedics, Guizhou Provincial People’s Hospital, Guiyang, China

**Keywords:** lumbosacral fixation and fusion, lumbopelvic fixation and fusion, sacroiliac joint, gait analysis, prospective cohort study

## Abstract

**Objective:**

To evaluate the effects of lumbosacral and lumbopelvic fixation and fusion on hip motor function using a three-dimensional gait analysis system.

**Methods:**

This single-center prospective cohort study enrolled adult patients who underwent lumbosacral or lumbopelvic fixation and fusion in the Orthopedics Department of Guizhou Provincial People's Hospital between November 2015 and January 2020. The patients were followed up postoperatively for over 1 year. During follow-up, the pain levels, functional impairment, and clinical outcomes of the patients were assessed using the Visual Analogue Scale score, Oswestry Disability Index, and Japanese Orthopedic Association score. Hip motor function was evaluated through a three-dimensional measurement system that assessed the hip, knee, and ankle joints. Data analysis was conducted using SPSS V22.0. This study was designed and reported to adhere to strengthening the reporting of observational studies in epidemiology (STROBE) guidelines for observational research.

**Results:**

A total of 30 patients were included in the study, with 15 undergoing lumbosacral prosthesis and 15 who underwent lumbopelvic fixation. The mean follow-up period was 13.8 months. A kinematic analysis revealed that, compared with healthy controls, patients who underwent lumbosacral fusion showed decreased minimum step length, maximum stride length, maximum angle, and range, while up-down and up-front displacements increased significantly (*p* < 0.05). Similar outcomes were observed in patients after lumbopelvic fixation and fusion, with an increase in maximum stride length compared with healthy controls (*p* < 0.05). When directly comparing the two groups, it was found that those who underwent lumbosacral fusion demonstrated greater maximum stride length, maximum hip flexion angle, and range of hip flexion and extension than those who underwent lumbopelvic fixation (*p* < 0.05).

**Conclusion:**

Both lumbosacral and lumbopelvic fixation and fusion significantly impact hip joint motor function compared with healthy controls. However, the increased mobility of the femoral head relative to the acetabulum in these patients may elevate the risk of hip osteoarthritis and cartilage wear. Within the scope of parameters measured in this gait analysis, patients receiving lumbosacral fixation demonstrated superiority in specific gait parameters, including maximum stride length and hip flexion–extension range of motion, compared with those who underwent lumbopelvic fixation. This suggests that preserving sacroiliac joint mobility may be beneficial for certain aspects of walking function when clinically justified.

## 1 Introduction

Lumbosacral pedicle screw fixation is the accepted operative approach for patients who require lumbosacral reconstruction, with satisfactory clinical effectiveness reported in the literature (1). In patients in whom enhanced biomechanical stability is needed, lumbosacral fixation is often combined with pelvic fixation (2). This combined lumbopelvic approach immobilizes the sacroiliac joints, which serve as a crucial link between the spine and the lower limbs. Consequently, mobility of the lumbar and sacral vertebrae, in addition to that of the pelvis, may be reduced, potentially affecting hip joint function. However, there is a scarcity of data comparing early functional outcomes between lumbosacral fixation alone and lumbopelvic fixation.

Previous studies in the field of lumbosacral fusion have established a foundation for various clinical scenarios. For instance, research on specific patient populations, such as those with lumbosacral agenesis, has introduced novel fusion methods (3), while percutaneous techniques for sacral fractures aim to reduce patient burden (4). Clear indications for lumbosacral fusion help clinicians make better treatment plans (5). Furthermore, studies have explored specific fixation methods such as bilateral iliac fixation and long-segment fusion criteria (4), and evaluated outcomes and complications in conditions such as lumbosacral tuberculosis (6) and tumors (7). Biomechanical comparisons of different techniques also provide a basis for optimization (8, 9).

Despite this body of work, current research lacks specific investigations into the impact of lumbosacral fixation and fusion on hip joint motor function. This study aims to address this gap, which is both necessary and innovative, as it can provide a better evidence base for clinical management.

Gait analysis serves as a key clinical tool for the quantitative assessment of gait abnormalities. It involves collecting detailed data on limb movement during walking, thereby informing diagnosis, treatment, and rehabilitation (10). However, traditional gait analysis methods, such as visual observation and two-dimensional (2D) analysis, have limitations. Visual observation is subjective and qualitative, lacking the ability to quantify gait parameters accurately (11). A simple 2D analysis fails to capture the three-dimensional (3D) nature of human gait, potentially leading to incomplete data. Therefore, this study utilizes the *Optimum* 3D motion measurement system (Shanghai InnoMotion) for gait assessment. This system employs infrared stereo tracking technology and multiple sensors to capture the 3D position of markers, providing precise motion data through advanced algorithms. Its high-precision sensors and multiparameter measurement capability make it suitable for detecting subtle changes in hip joint kinematics in postoperative patients, as supported by the technical principles and validation found in relevant literature (5, 11). The use of this advanced system ensures reliable and accurate data for evaluating the functional outcomes of the surgical techniques under investigation.

The primary aim of this study is to compare the effects of lumbosacral fixation and lumbopelvic fixation and fusion on hip joint motor function using a three-dimensional gait analysis system.

## 2 Materials and methods

### 2.1 Participants

This prospective cohort study included adult patients who underwent lumbosacral or lumbopelvic fixation and fusion for tuberculosis disease of the spine in the orthopedic ward of Guizhou Provincial People's Hospital between 1 November 2015 and 31 January 2020. All patients signed an informed consent form for inclusion, and this study was approved by our institutional Ethics Committee. Gait analyses from these patients were compared with those of a healthy control group for the exploration of any functional deficit.

The inclusion criteria for the study were as follows: (1) Patients who received lumbosacral or lumbopelvic fixation and fusion with at least 1 year of postoperative follow-up; (2) Postoperative radiology (CT/XR) suggestive of bony union of the graft without loosening or fracture of the internal fixation; (3) Patients who had made a satisfactory postoperative recovery; (4) Those who provided voluntary consent for study follow-up; (5) Those who were able to undergo three-dimensional gait analysis. A healthy control group was recruited, aged <40 years old, and with no significant comorbidities. All healthy controls had normal joint function of the lower limbs, with no past operative history or a history of trauma.

The exclusion criteria for the study were as follows: (1) Patients suffering from persistent dysfunction and/or pain in the lower back, sacroiliac joints, or lower limbs; (2) Patients with other comorbidities affecting spinal or limb mobility, including central nervous system diseases or ankylosing spondylitis; (3) Patients unable or unwilling to participate in gait analysis.

All procedures performed in studies involving human participants were in accordance with the ethical standards of the institutional and/or national research committee, and with the 1964 Helsinki Declaration and its later amendments, or comparable ethical standards. All participants provided informed consent before their participation in the study, and written consent was also obtained from all participants.

### 2.2 Research methods

Baseline characteristics, including gender, age, height, and weight, the Visual Analogue Scale (VAS) score, Oswestry Disability Index (ODI, also known as the Oswestry low back pain score), and Japanese Orthopedic Association (JOA) score at the last postoperative follow-up, were recorded for all included patients to evaluate their postoperative recovery and the ability to complete activities of daily living.

The three comparator groups (lumbosacral fusion vs. lumbopelvic fusion vs. healthy controls) underwent three-dimensional gait analysis. The effects of lumbosacral and lumbopelvic fixation and fusion on hip joint motor function were analyzed by comparing data across the three groups.

### 2.3 Three-dimensional gait analysis

**Figure 1:**
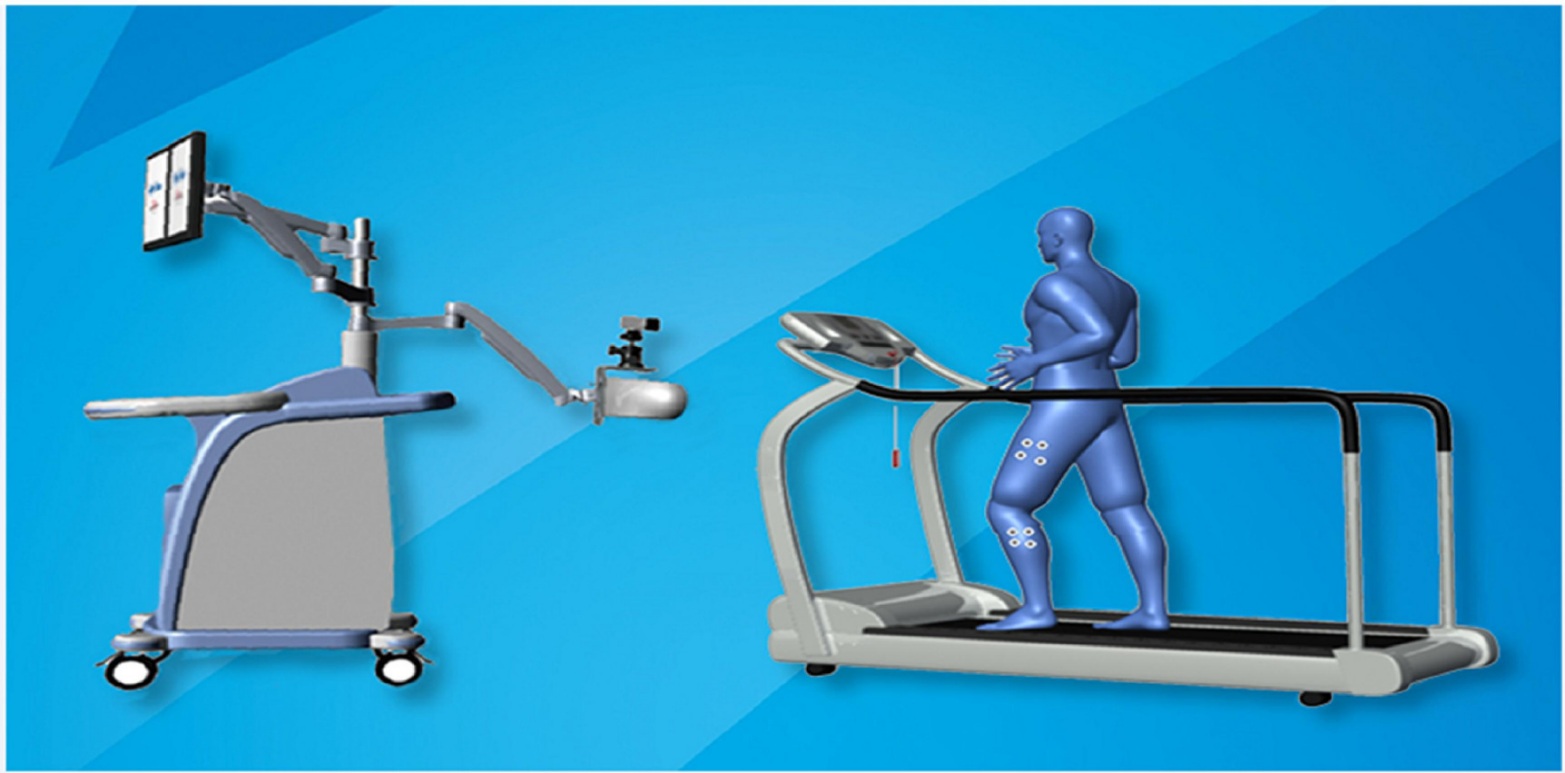
Schematic of the *Optimum* three-dimensional motion measurement system for the hip, knee, and ankle joints.

Three-dimensional gait analysis was completed at the Rehabilitation Center of the Guizhou Orthopedic Hospital using the *Optimum* three-dimensional motion measurement system for the hip, knee, and ankle joints (Shanghai InnoMotion, Figure 1). This system adopts infrared stereo tracking technology and calculates spatiotemporal, temporal, and motion parameters of the hip, knee, and ankle joints during walking. This works by capturing the three-dimensional position of marked points in real time. Before beginning the analysis, the study procedures and objectives were described in full to consenting patients. During the test, the subjects took off their shoes and were required to wear shorts. The position marker device was attached to the waist, one-third of the way down the lower thigh, one-third of the way down the upper calf, and on the dorsum of the foot; this ensured a direct line of sight between the infrared source and these markers and prevented patient movement. The bony surface landmarks (including the posterior superior iliac spine, anterior superior iliac spine, greater trochanter, lateral epicondyle of the femur, medial epicondyle of the femur, lateral malleolus, medial malleolus, heel, and second metatarsal head) of each subject were identified. First, the subjects ran on a treadmill at a speed of 3 km/h. Once the subjects got used to walking on the treadmill, the investigator confirmed that there was no disruption to the measurements recorded. Once stable, data collection was performed twice for each patient (approximately 15 s at a time). Following the completion of the gait analysis, the data were cleaned and transferred to statistical analysis software for processing.

The indicators under exploration included the step size, stride frequency, hip flexion and extension angles, the ranges of varus–valgus rotation, internal and external rotation, and motion during walking, and the ranges of forward–backward, upward–downward, and internal–external hip displacements.

### 2.4 Statistical analyses

Baseline demographic data were entered into a Microsoft Excel worksheet. After establishing the database, a statistical analysis was performed using SPSS software (V 22.0). Continuous data were first tested for normality using the Shapiro–Wilk test. Normally distributed data were presented as the mean ± standard deviation (SD) and were compared among the three groups using analysis of variance, with *post hoc* least significant difference tests applied where appropriate. Non-normally distributed data were expressed as median (interquartile range) and compared using the Mann–Whitney U test. Descriptive data were presented as percentages (%), and group comparisons were performed using the Chi-square test, with Fisher's exact test applied when appropriate. A two-sided *α* level of 0.05 was considered statistically significant.

## 3 Results

A total of 30 patients, 15 undergoing lumbosacral repair and 15 undergoing lumbopelvic repair, were included. In patients undergoing lumbosacral fixation and fusion, the distal extent of the fixation was the first sacral vertebra, and the proximal extent was the second lumbar vertebra in two patients, the third lumbar vertebra in eight, and the fourth lumbar vertebra in five. The distal extent of fixation for patients undergoing lumbopelvic fixation and fusion was the bilateral ilia, and the proximal extent was the second lumbar vertebra in four, the third lumbar vertebra in four, and the fourth lumbar vertebra in three. The mean length of follow-up was 13.8 months (range: 12–18 months). A total of 15 adult subjects were recruited as a healthy control group; of these, there were seven men, and eight were women.

No statistically significant differences were detected in the VAS, ODI, or JOA scores between the lumbosacral group and the lumbopelvic group at the final follow-up. The two fixation methods were able to achieve satisfactory clinical results (Table 1).

**Table 1:** Comparisons of functional outcomes between the two groups of patients at the time of final follow-up (mean ± SD).

Variable	Lumbosacral group	Lumbopelvic group	*p*
VAS	1.00 (0.50, 1.50)	1.00 (0.50, 1.50)	0.626
ODI	2.00 (1.00, 5.00)	3.00 (1.00, 3.00)	0.951
JOA	25.60 ± 3.25	24.20 ± 2.83	0.219

There were no differences in gender, height, weight, or BMI among the control, the lumbosacral fusion, and the lumbopelvic fusion groups. However, the patients in the control group were significantly younger than those in the other groups (Table 2).

**Table 2:** Comparisons of baseline characteristics across the three groups (mean ± SD).

Variable	Group	Control group	Lumbosacral group	Lumbopelvic group	*p*
Gender	Male subjects	7 (46.7%)	7 (46.7%)	8 (53.3%)	0.915
	Female subjects	8 (53.3%)	8 (53.3%)	7 (46.7%)	
Age (years old)		25.47 ± 1.30^a^	34.67 ± 10.49^b^	33.87 ± 10.36^b^	0.009
Height (cm)		163.87 ± 8.72	163.80 ± 8.44	162.93 ± 10.67	0.954
Weight (kg)		59.33 ± 9.72	60.13 ± 8.88	59.47 ± 10.09	0.970
Body mass index (BMI) (kg/m^2^)		22.02 ± 2.18	22.42 ± 2.80	22.63 ± 2.83	0.816

Groups marked with the same letter had no statistically significant differences (*p* > 0.05), but those marked with different letters had statistically significant differences (*p* < 0.05).

The results of the gait analysis for the three comparator groups are presented in Table 3. The minimum step size was significantly smaller in the lumbosacral and lumbopelvic groups than in the healthy controls (*p* = 0.01). However, there was no significant difference in this aspect between the lumbosacral and the lumbopelvic groups. Similarly, significant differences were seen in the maximum step size among the three comparator groups. The minimum stride frequency was marginally higher in the lumbosacral and lumbopelvic groups than in the control group, but this was not statistically significant. However, while there was no significant difference in the maximum stride frequency between the control and the lumbosacral groups, nor between the lumbosacral and the lumbopelvic groups, there was a statistically significant difference between the control and the lumbopelvic groups.

**Table 3:** Comparisons of step size (cm) and stride frequency (n/s) across the three groups (mean ± SD).

Variable	Control group	Lumbosacral group	Lumbopelvic group	*p*
Min (step size)	45.45 ± 6.55^a^	41.21 ± 4.50^b^	39.55 ± 4.24^b^	0.010
Max (step size)	50.33 ± 5.68^a^	46.17 ± 4.46^b^	42.54 ± 2.65^c^	<0.001
Min (stride frequency)	1.57 ± 0.15	1.65 ± 0.14	1.73 ± 0.20	0.051
Max (stride frequency)	1.76 ± 0.16^a^	1.89 ± 0.14^a,b^	1.94 ± 0.24^b^	0.033

Groups marked with the same letter had no statistically significant differences (*p* > 0.05), but those marked with different letters had statistically significant differences (*p* < 0.05).

A comparison of the profiles of movement among the three groups can be found in Table 4. The maximum hip flexion and extension angles and their maximum ranges were significantly lower in the lumbosacral and lumbopelvic groups than in healthy controls. In contrast, there were no statistically significant differences in the minimum hip flexion and extension angles, varus–valgus rotation, or internal and external rotation among the three groups.

**Table 4:** Comparisons of the angles of flexion and extension, varus–valgus rotation, and internal and external rotation of hip joints across the three groups (°, mean ± SD).

Variable	Control group	Lumbosacral group	Lumbopelvic group	*p*
Min (hip flexion and extension angles)	−7.95 ± 1.59	−7.83 ± 1.44	−7.66 ± 2.36	0.908
Max (hip flexion and extension angles)	38.85 ± 1.97^a^	36.55 ± 3.01^b^	33.95 ± 2.30^c^	0.002
Hip flexion and extension angles during forward walking (FW)	46.81 ± 2.75^a^	44.38 ± 3.10^b^	41.61 ± 3.46^c^	<0.001
Min (varus–valgus hip rotation angle)	−4.36 ± 0.75	−4.06 ± 0.85	−4.27 ± 1.61	0.761
Max (hip varus–valgus rotation angle)	3.98 ± 0.54	3.91 ± 0.54	3.77 ± 1.18	0.777
Hip varus–valgus rotation angle during FW	8.34 ± 1.24	7.99 ± 1.10	8.04 ± 1.91	0.776
Min (hip internal and external rotation angles)	−5.17 ± 1.23	−5.19 ± 0.66	−5.07 ± 0.56	0.917
Max (hip internal and external rotation angles)	6.39 ± 1.21	6.18 ± 0.42	6.25 ± 0.99	0.829
Hip internal and external rotation angles during FW	11.56 ± 1.17	11.37 ± 0.91	11.24 ± 1.01	0.698

Groups marked with the same letter had no statistically significant differences (*p* > 0.05), but those marked with different letters had statistically significant differences (*p* < 0.05).

Summary data on the displacement of the hip joint in the three groups are provided in Table 5. The maximum values and ranges of forward–backward and upward–downward hip displacements were significantly higher in the lumbosacral and lumbopelvic groups than in healthy controls. However, there were no significant differences in the values between the lumbosacral and the lumbopelvic groups. There were no significant differences in the minimum values of forward–backward, upward–downward hip displacements, and internal–external hip displacement among the three groups.

**Table 5:** Comparisons of forward–backward, upward–downward, and internal–external hip displacements across the three groups (cm, mean ± SD).

Variable	Control group	Lumbosacral group	Lumbopelvic group	*p*
Min (forward–backward hip displacement)	−0.91 ± 0.37	−1.03 ± 0.44	−0.99 ± 0.35	0.688
Max (forward–backward hip displacement)	0.89 ± 0.60^a^	1.43 ± 0.42^b^	1.49 ± 0.44^b^	0.003
Forward–backward hip displacement during FW	1.80 ± 0.47^a^	2.46 ± 0.40^b^	2.48 ± 0.41^b^	<0.001
Min (upward–downward hip displacement)	−0.69 ± 0.55	−0.82 ± 0.42	−0.85 ± 0.44	0.642
Max (upward–downward hip displacement)	1.00 ± 0.41^a^	1.59 ± 0.50^b^	1.63 ± 0.67^b^	0.004
Upward–downward hip displacement during FW	1.69 ± 0.40^a^	2.42 ± 0.38^b^	2.47 ± 0.63^b^	<0.001
Min (internal–external hip displacement)	−0.96 ± 0.36	−0.89 ± 0.46	−0.90 ± 0.35	0.879
Max (internal–external hip displacement)	0.87 ± 0.39	0.91 ± 0.41	0.94 ± 0.32	0.888
Internal–external hip displacement during FW	1.83 ± 0.22	1.81 ± 0.36	1.84 ± 0.33	0.952

Min represents the minimum value, Max represents the maximum value, and FW represents the range of activities.

Groups marked with the same letter had no statistically significant differences (*p* > 0.05), but those marked with different letters had statistically significant differences (*p* < 0.05).

## 4 Discussion

Recent research has proposed that the motor coordination of the spine–pelvis–hip joint balances both the mass of the trunk and the hip motion during standing and walking (12). Any dysfunction along this kinematic chain will result in biomechanical changes that can have adverse effects on adjacent components. Thus, restriction of the motion of the lumbar spine and pelvis (as in the case of lumbar fixation and fusion) may affect the overall function of this spine–pelvis–hip joint unit. Crucially, this integrated coordination, often described as the “spinopelvic rhythm,” ensures efficient force transmission and moment equilibrium during gait. Disruption of this rhythm alters the mechanical demands on adjacent segments.

“Gait” refers to the body posture when walking. A healthy population without spinal disease shares common components in their gaits, including cyclic and alternate swing. This reciprocating movement enables researchers to study changes in gait in the presence of disease (13).

### 4.1 Research on step size and stride frequency

Step size refers to the average distance from one heel to the other, and stride frequency is the mean number of steps per second or minute (n/s, n/min). At an autonomous pace, step size and stride frequency during walking change with gender, age, and height (14). In this study, two groups of subjects who had undergone lumbosacral or lumbopelvic fixation and fusion with a similar distribution of gender, age, and height were selected and matched to a group of healthy controls to allow fair comparison. However, as shown in Table 2, the healthy control group was significantly younger than the two surgical groups. Because age is a well-established factor influencing gait parameters and hip joint biomechanics, and is with advanced age often associated with natural degenerative changes, this age mismatch may introduce confounding bias and limit the direct comparability of gait outcomes across groups. However, because all subjects in this study were either young or middle-aged, they had largely comparable anticipated gait characteristics (15).

The step size in the lumbosacral group and lumbopelvic group was smaller than that in the control group. This is likely due to the effect of lumbosacral fusion increasing stiffness and reducing the three-dimensional mobility of the lumbar spine, which restricts the motion of the trunk during walking. In the lumbosacral group, lumbosacral fusion also restricted the motion of the lumbar spine and pelvis to some extent. This was seen as a reduction in the rotation angle of the horizontal pelvic plane (forward and backward motion). In addition, the extension of the hip joints was insufficient at the end of the support phase in this group, and hip flexion was insufficient at the end of the swing phase. Together, these contributed to a reduction in step size. In this study, subjects were tested at a constant speed of 3 km/h, and therefore, the stride frequency in both the lumbosacral and the lumbopelvic groups often increased to compensate for a loss of step speed where the step size was shortened. The increased stride frequency accelerated the cyclic change of the gait of these patients. As each gait cycle requires energy for muscle contraction, this is likely to increase energy consumption from walking and decrease step efficiency.

### 4.2 Hip joint mobility

The standard movements at the hip joint are flexion and extension, varus–valgus rotation, and internal and external rotation. In both the lumbosacral and lumbopelvic groups, the maximum angles and ranges of hip flexion and extension were significantly smaller than those in the control group. During walking, the center of gravity of the trunk moves along with the line of gravity of the lower limbs, causing a slight extension of the lumbar spine with anteversion. This activity of the lumbar vertebrae largely occurs in the areas affected by lumbosacral fusion, including the fourth/fifth lumbar vertebra segment, the fifth lumbar vertebra, and the first sacral spine segment. The activities of these lumbar vertebrae are limited by fusion, and the biomechanics of the lower limbs, therefore, change. This may, for example, reduce the angles of hip flexion and extension. Lumbopelvic fixation and fusion simultaneously decrease the activity of the lumbar vertebra and pelvis, which will markedly affect the motor function of the lower limbs. During walking, at the end of the swing phase and initial landing, the pelvis has a horizontal 5° forward rotation, which is key to lower limb flexion. At the end of the support phase, the pelvis demonstrates a 5° backward rotation, contributing to lower limb extension. These functions are very important for determining step size in humans. After lumbosacral fusion, however, pelvic motion declines or even disappears, which weakens flexion and extension of the lower limbs. This manifests as a reduction in the flexion and extension angles of the hip joints. As flexion and extension at the hip joints are the primary determinants of step size during walking, step size will be decreased when these movements are restricted, thus further affecting step speed. This is consistent with the reduction in step size seen in this study.

The flexion and extension of the hip joints closely correlate with the range of motion of the sacroiliac joints. The sacroiliac joints, located in the posterior pelvic ring, are a crucial part of gait biomechanics, although they have a limited range of movement even in health. In gait cyclic motions, the sacroiliac joints have an independent impact on both the stability and the motor function of the lower limbs. Previous measurements of the full motion range of sacroiliac joints indicate that the maximum motion range of sacroiliac joints usually occurs under conditions of extreme flexion and extension of hip joints (16, 17). This may be the reason why the maximum hip flexion and extension angles decreased but showed no differences among the three groups in this study. In the present study, the angles and ranges of hip varus–valgus rotation and internal and external rotation were not significantly different across the three groups. This may be explained as the hip allowing extra motions within its normal range, with a relatively small range of varus–valgus rotation when walking.

### 4.3 Hip displacement

Hip displacement is defined as the movement of the femoral head relative to the acetabulum. In this study, we revealed that the maximum values and ranges of forward–backward and upward–downward hip displacements after both lumbosacral and lumbopelvic fixation and fusion were higher in the two surgical groups than in healthy controls; this indicates increased motion of the femoral head relative to the acetabulum. However, there were no differences in the values between the lumbosacral and the lumbopelvic groups.

This key finding can be interpreted within the “spinopelvic rhythm” framework. Loss of lumbar and sacroiliac joint mobility disrupts the normal sagittal plane moment equilibrium maintained by the coordinated movement of the spine, pelvis, and hips. To compensate for the restricted pelvic rotation and maintain forward propulsion, the hip joint may be forced to generate greater sagittal plane translation. This compensatory mechanism, where the femoral head exhibits increased anterior–posterior and superior–inferior glide within the acetabulum, acts to generate the necessary moment to substitute for the lost pelvic contribution. It is a direct biomechanical consequence of the altered spinopelvic rhythm, rather than solely a change in the osseous position of the acetabulum.

In clinical practice, hip joint pain can occur in some patients after long-segment spinal fixation and fusion. This is likely due to a change in the position of the acetabulum relative to the femoral head postoperatively. This reduces the coverage of the acetabulum, reducing hip stability and potentially accelerating long-term hip joint degeneration (18). Several recent studies have reported that lumbar fusion increases the risk of dislocation and the need for total hip replacement revision (19, 20). This may be caused by decreased pelvic inclination related to reduced lumbar flexibility after lumbar fusion. Flexible lumbosacral units increase the stability of total hip replacements and avoid impact on and dislocation of prostheses. This protective effect can be eliminated after lumbar fusion (21).

Research on the biomechanical coordination between the spine and the hip joints has suggested that every 1° increase in pelvic retroversion increases acetabular anteversion by 0.7° (22). When moving from standing to sitting, lumbar lordosis declines, the sacrum and pelvis retrovert, and the acetabular anteversion increases to adapt to the bent femur to prevent stress and dislocation (23). When the hip joint flexes after lumbosacral fusion, the sacrum can no longer retrovert, and normal acetabular anteversion declines. This may lead to the movement of the femoral head to the edge of the acetabulum, potentially increasing stress across the hip joint, which could predispose to dislocation (24). The hip joint, composed of the acetabulum and the femoral head, is a ball-and-socket joint. The acetabulum is a part of the pelvis, and when its motion changes, the position of the acetabulum relative to that of the femoral head also changes. This results in changes to hip displacement. An analysis of this segment has demonstrated significant changes in biomechanics with the changes in lumbar lordosis and the inclination of the sacrum and pelvis. Lumbosacral and lumbopelvic fixation, therefore, puts stress on adjacent parts, which influences the position of the acetabulum relative to the femoral head. The observed increase in hip displacement likely represents a composite of these osseous alignment changes and the aforementioned dynamic compensatory mechanism within the altered spinopelvic rhythm.

However, the exact mechanism remains to be further clarified. In this study, it was found that only the forward–backward and upward–downward displacements increased after fixation, with no clear differences in left and right displacements. The predominance of sagittal plane (forward–backward and upward–downward) displacements aligns with the theory of compensation for lost sagittal plane pelvic motion and moment equilibrium.

While our analysis demonstrated that lumbosacral and lumbopelvic fixation affects hip joint motion, no obvious difference could be seen when observing the walking gait of the three groups of patients with the naked eye; a detailed comparison was facilitated by formal gait analysis techniques. In addition, all patients had satisfactory levels of function at their final follow-up appointment and did not report any ongoing pain or discomfort. The altered biomechanics and increased hip displacement observed in this study represent potential risk factors for long-term joint degeneration. However, this study provides no direct data on cartilage wear or long-term prognosis. Therefore, the long-term clinical impact of these subtle changes in gait, including their potential role in the development of hip osteoarthritis, requires further exploration. Future research should directly investigate hip joint degeneration through longer-term follow-ups and dedicated imaging assessments (e.g., MRI).

This study on the influence of lumbosacral and lumbopelvic fixation and fusion on hip joint motor function should be considered preliminary. Several limitations should be acknowledged. First, the healthy control group was significantly younger than the surgical groups. Because age is a known determinant of gait kinematics and hip joint integrity, this mismatch may have confounded the comparison of gait parameters and hip displacement across the groups. The younger controls may have exhibited better-preserved joint mobility and muscle function, which could have amplified the observed differences. Future studies should implement stricter age-matching to minimize this potential bias. The lower limbs act as a chain-like structure involving interlinking components, including the hip, knee, and ankle joints. To comprehensively assess the effects of lumbar fixation and fusion on lower limb motor function, it will be necessary to examine compensatory changes in both the knee joints and the ankle joints as hip joint motion changes.

Within the limited sample size of this study, we observed that patients undergoing lumbosacral fusion demonstrated superiority over the lumbopelvic fusion group in certain gait parameters. This finding warrants further validation in larger-scale studies.

## Data Availability

The original contributions presented in the study are included in the article/Supplementary Material, and further inquiries can be directed to the corresponding authors.

## References

[1] Dong S Tang X Ji T Guo W. The spinopelvic reconstruction after total sacrectomy. Chin J Bone Joint Surg. (2018) 11(07):481–5. 10.3969/j.issn.2095-9958.2018.07.001

[2] Joukar A Mehta J Goel VK Marks DS. Biomechanical analysis of the tuning fork plate versus dual pelvic screws in a sacrectomy model: a finite element study. Global Spine J. (2021) 12(7):1495–502. 10.1177/2192568220983792 33517788 PMC9393982

[3] Yazici M Akel I Demirkiran HG. Lumbopelvic fusion with a new fixation technique in lumbosacral agenesis: three cases. J Child Orthop. (2011) 5(1):55–61. 10.1007/s11832-010-0322-2 22295050 PMC3024487

[4] McCord DH Cunningham BW Shono Y Myers JJ McAfee PC. Biomechanical analysis of lumbosacral fixation. Spine (Phila Pa 1976). (1992) 17(8 Suppl):S235–43. 10.1097/00007632-199208001-00004 1523506

[5] Williams SK Quinnan SM. Percutaneous lumbopelvic fixation for reduction and stabilization of sacral fractures with spinopelvic dissociation patterns. J Orthop Trauma. (2016) 30(9):e318–24. 10.1097/BOT.0000000000000559 26894767 PMC4987022

[6] Yu L Li L Li D Luo Z Liu N Wu Y Surgical strategy of lumbopelvic instrumentation in the treatment of lumbosacral tuberculosis: S2-alar-iliac screws vs iliac screws. Int Wound J. (2022) 19(8):1964–74. 10.1111/iwj.13795 35297177 PMC9705182

[7] Jackson RJ Gokaslan ZL. Spinal-pelvic fixation in patients with lumbosacral neoplasms. J Neurosurg. (2000) 92(1 Suppl):61–70. 10616060 10.3171/spi.2000.92.1.0061

[8] McCord DH Cunningham BW Shono Y Myers JJ McAfee PC. Biomechanical analysis of lumbosacral fixation. In: Yonenobu K Ono K Takemitsu Y, editors. Lumbar Fusion and Stabilization. Tokyo: Springer (1993) 17(8 Suppl):S235–43. 10.1097/00007632-199208001-00004 1523506

[9] Leong JC Lu WW Zheng Y Zhu Q Zhong S. Comparison of the strengths of lumbosacral fixation achieved with techniques using one and two triangulated sacral screws. Spine. (1998) 21(1):11–5. 10.1097/00007632-199811010-00008 9820908

[10] Xu L-y Zhou Y Liu M-k Lin L Chen Q-q Li H-y. Effects of mirror therapy on motor function of lower extremities and walking in subacute stroke patients. Chin J Rehabil Theory Pract. (2018) 4(07):834–8. 10.3969/J.ISSN.1006-9771.2018.07.015

[11] Hutabarat Y Owaki D Hayashibe M. Recent advances in quantitative gait analysis using wearable sensors: a review. IEEE Sens J. (2021) 21(23):26470–87. 10.1109/JSEN.2021.3119658

[12] Ike H Dorr LD Trasolini N Stefl M McKnight B Heckmann N. Spine-pelvis-hip relationship in the functioning of a total hip replacement. J Bone Joint Surg. (2018) 100(18):1606–15. 10.2106/JBJS.17.00403 30234627

[13] Wang W Tian F Li N Li G Cai Z Wang S. Development and clinical application of novel motion analysis system for knee. Chin J Joint Surg. (2020) 14(01):78–84. 10.3877/cma.j.issn.1674-134X.2020.01.014

[14] Jafarnezhadgero AA Pourrahimghoroghchi A Darvishani MA Aali S Dionisio VC. Analysis of ground reaction forces and muscle activity in individuals with anterior cruciate ligament reconstruction during different running strike patterns. Gait Posture. (2021) 90:204–9. 10.1016/j.gaitpost.2021.09.167 34509043

[15] Li X Shu B Gu X Jiang W Lv L. Temporal-spatial parameters of gait: reference data of normal subjects from Chinese adults. Chin J Rehabil Med. (2012) 27(03):227–30. 10.3969/j.issn.1001-1242.2012.03.008

[16] Hammer N Scholze M Kibsgård T Klima S Schleifenbaum S Seidel T Physiological *in vitro* sacroiliac joint motion: a study on three-dimensional posterior pelvic ring kinematics. J Anat. (2019) 234(3):346–58. 10.1111/joa.12924 30536830 PMC6365483

[17] Adhia DB Milosavljevic S Tumilty S Bussey MD. Innominate movement patterns, rotation trends and range of motion in individuals with low back pain of sacroiliac joint origin. Man Ther. (2016) 21:100–8. 10.1016/j.math.2015.06.004 26144685

[18] Si G Li T Liu X Liu Z Li W Yu M. Correlation analysis between postoperative hip pain and spino-pelvic-hip parameters in adult scoliosis patients after long-segment spinal fusion. Chin J Spine Spinal Cord. (2018) 28(11):989–94. 10.3969/j.issn.1004-406X.2018.11.05 32006111

[19] Malkani AL Garber AT Ong KL Dimar JR Baykal D Glassman SD Total hip arthroplasty in patients with previous lumbar fusion surgery: are there more dislocations and revisions? J Arthroplasty. (2018) 33(4):1189–93. 10.1016/j.arth.2017.10.041 29174406

[20] Salib CG Reina N Perry KI Taunton MJ Berry DJ Abdel MP. Lumbar fusion involving the sacrum increases dislocation risk in primary total hip arthroplasty. Bone Joint J. (2019) 101-B(2):198–206. 10.1302/0301-620X.101B2.BJJ-2018-0754.R1 30700125

[21] Buckland AJ Puvanesarajah V Vigdorchik J Schwarzkopf R Jain A Klineberg EO Dislocation of primary total hip arthroplasty is more common in patients with lumbar spinal fusion. Spine J. (2016) 16(10):S263–4. 10.1016/j.spinee.2016.07.356 28455466

[22] Yun HH Murphy WS Ward DM Zheng G Hayden B Murphy SB. Effect of pelvic tilt and rotation on cup orientation in standing anteroposterior radiographs. Hip Int. (2020) 30(1):48–55. 10.1177/1120700019831665 30834795

[23] Radcliff KE Kepler CK Hellman M Restrepo C Jung KA Vaccaro AR Does spinal alignment influence acetabular orientation: a study of spinopelvic variables and sagittal acetabular version. Orthop Surg. (2014) 6(1):15–22. 10.1111/os.12090 24590988 PMC6583444

[24] Lum ZC Coury JG Cohen JL Dorr LD. The current knowledge on spinopelvic mobility. J Arthroplasty. (2017) 33(1):291–6. 10.1016/j.arth.2017.08.013 28939031

